# Classification and Bundle‐Weaving Fixation for Posterior Cruciate Ligament Tibial Avulsion Fractures: Innovations and Applications of Key Techniques

**DOI:** 10.1111/os.70135

**Published:** 2025-08-29

**Authors:** Guangdong Chen, Weiguo Xu, Yibing Chen, Wei Wang, Chengyue Yu

**Affiliations:** ^1^ Academy of Medical Engineering and Translational Medicine Tianjin University Tianjin China; ^2^ Department of Neurosurgery UCSF Medical Center San Francisco California USA; ^3^ Department of Orthopedics Cangzhou Central Hospital Cangzhou China; ^4^ Tianjin Hospital, Tianjin University Tianjin China; ^5^ Queen Mary School, Jiangxi Medical College Nanchang University Nanchang China; ^6^ Department of Research Shurun Health Technology (Hebei) Co., Ltd. Cangzhou China; ^7^ Department of Rehabilitation Tianjin Hospital, Tianjin University Tianjin China

**Keywords:** 3D‐printed guide plate, avulsion fracture, customized fixation method, fracture classification, novel fixation system with tendon‐weaving holes

## Abstract

**Objective:**

Posterior cruciate ligament tibial avulsion fractures (PCLTAF) are characterized by complex injury mechanisms and treatment difficulties, with no standardized diagnostic or therapeutic guidelines currently available. This study aims to establish a classification‐based Bundle‐Weaving Zonal Fixation system to facilitate the precise treatment of PCLTAF.

**Methods:**

A retrospective analysis was conducted on 100 patients with PCLTAF treated across multiple centers between 2016 and 2022. Based on fracture morphology, fragment characteristics, bone quality, and the extent of posterior ligamentous complex involvement, a novel classification system—Xu‐Chen concise classification—was developed using an inductive approach, categorizing PCLTAF into nine subtypes. Guided by this classification, four bundle‐weaving fixation techniques were designed, along with the development of a novel fixation system with tendon‐weaving holes. One representative case from each subtype (*n* = 9) underwent open reduction and subtype‐guided individualized fixation. Surgical time, intraoperative blood loss, complications, fracture healing, and functional outcomes (Lysholm and Tegner scores) were assessed.

**Results:**

According to the Xu‐Chen concise classification, nine patients (six males and three females; mean age, 45 years) underwent open reduction and individualized bundle‐weaving fixation. The average surgical duration was 77.2 min (range, 60–95 min), and the average intraoperative blood loss was 23.3 mL (range, 15–40 mL). The mean follow‐up period was 17.89 months (range, 12–22 months). The Lysholm knee score improved from 26.78 preoperatively to 97.22 postoperatively, while the Tegner activity score increased from 2.89 to 9.56. No complications such as deep vein thrombosis, joint stiffness, postoperative swelling, hematoma, infection, fixation failure, joint instability, or refracture were observed during follow‐up. Only one elderly patient experienced superficial wound necrosis, which healed with conservative wound care.

**Conclusion:**

The Xu‐Chen concise classification and its corresponding tendon‐bundled intraosseous fixation system offer a structured and standardized treatment pathway for PCLTAF. Preliminary results demonstrate promising outcomes in anatomical reduction, functional recovery, and surgical safety. This strategy shows clinical value in managing complex cases, such as comminuted fractures and osteoporotic bone.

## Graphical abstract

This study proposes a new classification method for posterior cruciate ligament tibia avulsion fracture and introduces a personalized bundle‐weaving fixation technique, achieving precise reduction and improved functional recovery, especially for complex fractures, making it a safe and effective treatment strategy.

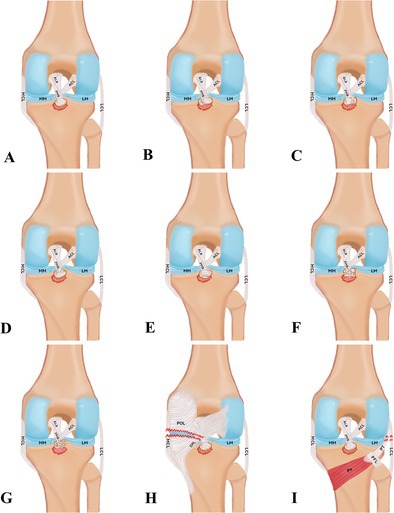

## 1 Introduction

Posterior cruciate ligament tibial avulsion fracture (PCLTAF) is a type of injury that severely affects knee joint function, often leading to posterior and rotational instability, thereby accelerating degenerative changes in the knee joint. Currently, standardized diagnosis and treatment of such fractures remain a clinical challenge.

Studies have shown that the injury mechanism of PCLTAF is usually caused by a direct force pushing the proximal tibia posteriorly when the knee is flexed or by excessive knee hyperextension [1]. Low‐energy trauma often leads to tears in the mid‐substance of PCL, whereas high‐energy injuries are more likely to result in avulsion fractures [2]. Since part of the posterior cruciate ligament tibial attachment is located outside the joint capsule, avulsion fractures often result in bone fragments being embedded within the joint capsule and surrounding soft tissues. Most patients with PCL injuries require surgical treatment [3, 4], and those who do not undergo surgery often fail to regain their pre‐injury level of athletic performance or functional recovery [5].

Traditional classification methods, such as the Meyers and McKeever classification [6] and the evaluation criteria proposed by Harner et al. [7], do not provide a systematic and comprehensive classification of fractures. These methods lack quantitative assessment of fracture fragments and fail to adequately consider important associated injuries, including periligamentous complex injuries [8], meniscal damage [1], and reverse Segond fractures [9]. Therefore, their clinical utility in guiding precise surgical planning is limited.

In PCLTAF treatment, traditional surgical fixation materials such as cannulated screws, suture anchors, and Endobutton often suffer from inadequate early fixation stability, leading to prolonged postoperative rehabilitation, joint stiffness, and even failure of elastic fixation. In recent years, an increasing number of studies have reported the use of plate‐based fixation techniques [10, 11, 12, 13, 14], primarily utilizing 1/3 semi‐tubular plates or 3.5 mm suture anchor augmentation. However, due to the anatomical complexity of the PCL attachment site, the application of standardized plates remains significantly limited. A study by Liu [15] demonstrated that, compared to younger patients, those with osteoporosis or advanced age have higher demands for fixation stability. Currently available treatment strategies struggle to simultaneously address both the heterogeneity of fracture fragments and the associated soft tissue injuries, lacking a systematic and standardized diagnostic and therapeutic framework. Given these challenges, achieving precision treatment within modern surgical paradigms has become a pressing issue in orthopedics.

This study is the first to propose: (1) The Xu‐Chen concise classification, which mechanistically and systematically categorizes PCLTAF, accurately identifying the morphological features and surgical indications of each injury type. This classification is structurally clear, practical, and easy to apply, addressing the limitations of traditional systems such as ambiguous decision‐making pathways and insufficient surgical guidance. It enables surgeons to rapidly identify cases requiring intervention. (2) Based on the Xu‐Chen concise classification, four corresponding bundle‐weaving fixation techniques were developed, forming an integrated “classification‐guided, bundle‐weaving anchoring” decision‐making framework. This approach comprehensively covers various PCLTAF injury patterns, significantly enhances the early strength and stability of PCL reconstruction, and effectively resolves key issues in complex cases such as inconsistent surgical planning and the lack of classification‐based guidance. (3) Since 2016, our team has developed three generations of internal fixation devices for PCLTAF management: First‐generation: A novel fixation system with tendon‐weaving holes (Patent No. ZL201620025670.3), designed to precisely fit the irregular and posteriorly inclined surface of the tibial plateau. Second‐generation: An optimized tendon‐weaving anchor hole configuration featuring a single‐row of four semi‐threaded gourd‐shaped holes, connected in pairs. This design accommodates both suture weaving and screw insertion, improving fixation feasibility for comminuted proximal fractures. Third‐generation: Introduction of a “protrusion” structure with a breakpoint design, integrating multi‐axial screw channels and ligament‐weaving anchor holes. Anatomically contoured plates were developed for the right (TC‐R) and left (TC‐L) sides, enabling multi‐angled directional compression fixation at the ligament insertion site. This generation significantly improves surgical safety, intraoperative flexibility, and individualized adaptability, offering a comprehensive and innovative solution for the clinical management of PCLTAF.

## 2 Patients and Methods

### 2.1 Classification and Patient Distribution Characteristics

This study retrospectively analyzed 100 cases of patients who underwent treatment for tibial avulsion fractures at the posterior cruciate ligament (PCL) insertion site between May 2016 and May 2022 across multiple medical centers.

### 2.2 Imaging Acquisition and Analysis

All patients underwent both knee CT and MRI examinations. Case images were screened using 3D reconstruction from CT scans. Fracture lines and displacement of avulsed bone fragments were further assessed using axial, coronal, and sagittal views (decision interval of 3–5 mm). The degree of displacement was evaluated by measuring the linear distance between the fracture end and the corresponding cortical reference point, with the maximum value recorded as the result. MRI was used to assess signal changes indicative of injuries to the posterior ligamentous complex and other key soft tissue structures.

### 2.3 Classification Method Development and Implementation

The research team developed a new PCLTAF classification system based on imaging characteristics and clinical manifestations, using a manually inductive method guided by anatomical orientation, clinical experience, and expert consensus. The classification process included: (1) assessing the integrity of the avulsed fragment; (2) for bipartite fragments, recording the anatomical orientation (medial, lateral, superior, inferior) of the minor fragment avulsion; (3) special types, such as quadrant‐shaped fragments or those with completely comminuted attachment sites, were recorded separately; (4) MRI‐indicated partial or complete fiber rupture of the posterior ligamentous complex was also marked. This classification comprehensively considers the morphology, size, and distribution of the avulsed fragment, bone quality (e.g., osteoporosis), and involvement of the posterior ligamentous complex. All cases were independently classified by two senior orthopedic surgeons under blinded conditions. A third expert then reviewed and confirmed the results to resolve discrepancies and finalize the classification, ensuring consistency and data integrity.

### 2.4 Methodological Advantages

This classification relies entirely on directly visualized imaging features, ensuring high objectivity and operational stability. The use of a “double‐blind review with third‐party adjudication” process ensures data consistency without the need for further inter‐observer agreement statistical analysis. This strategy enhances efficiency while maintaining scientific rigor and reproducibility, meeting the study's requirements for practical applicability and clinical scalability.

### 2.5 PCLTAF Classification Nomenclature and Case Selection Criteria

**FIGURE 1:**
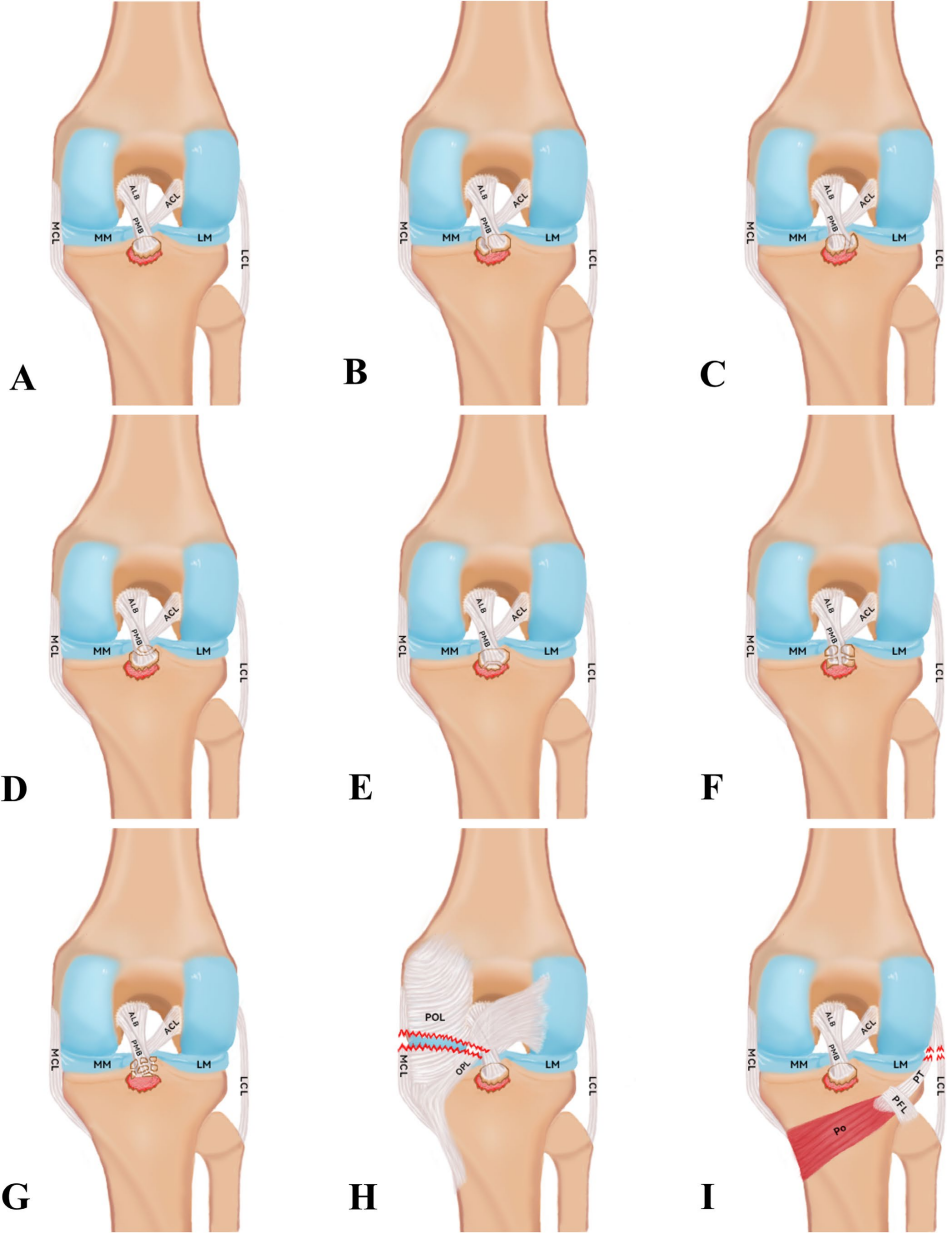
Xu‐Chen concise classification of PCLTAF.

Named after the surnames of the primary investigators, this system is referred to as the Xu‐Chen concise classification of fractures. According to this system, PCLTAFs are categorized into nine distinct types: Type‐A:Monoblock Type, Type‐B:Medial Dominant Split Type, Type‐C:Lateral Dominant Split Type, Type‐D:Proximal Dominant Split Type, Type‐E: Distal Dominant Split Type, Type‐F:Quadrant Comminution Type, Type‐G:Complete Footprint Comminution Type, Type‐H:Fracture with Medial Ligament Complex Type, Type‐I:Fracture with Lateral Ligament Complex Type (Figure 1). Among the 100 patients with PCLTAF, 4 were classified as Type A, 2 as Type B, 3 as Type C, and 1 as Type H. Imaging revealed PCLTAF without significant displacement or suggestive of ligament rupture; therefore, non‐operative treatment was selected based on individualized clinical evaluation. Detailed data distribution characteristics are presented (Table 1). Inclusion Criteria: (1) Patients diagnosed with PCLTAF confirmed by X‐ray, CT, or MRI; (2) Age ≥ 18 years, regardless of sex; (3) Surgical treatment performed within 3 weeks of injury; (4) Willingness to undergo individualized classification‐based bundle‐weaving fixation and complete standardized follow‐up; (5) Ability to understand and sign the informed consent form and willingness to comply with clinical pathway management. Exclusion Criteria: (1) Severe concomitant injuries to the ACL; (2) Associated comminuted tibial plateau fractures or neurovascular injuries that interfere with surgical fixation; (3) History of prior surgery on the ipsilateral knee or chronic PCL injury with fracture; (4) Active infection, rheumatic disease, malignancy, or severe osteoporosis that precludes adequate fixation; (5) Psychiatric disorders, impaired consciousness, or other conditions preventing cooperation with surgery and follow‐up; (6) Refusal to participate in the study protocol or loss to follow‐up postoperatively.

**TABLE 1:** Distribution characteristics of Xu‐Chen concise classification in 100 PCLTAF cases.

Number of Patients	Classification	Sex		Age (years)	BMI (Kg/m^2^)	History of trauma		Bone mineral density (SD)	Treatment method	
		M	F			Traffic injury	Sports injury		Non‐Surgical	Surgical
30	Type‐A	21	9	37.37 ± 10.84	27.61 ± 2.45	10	20	−0.71 ± 0.61	4	26
8	Type‐B	5	3	42.13 ± 11.37	29 ± 1.86	1	7	−1.04 ± 0.51	2	6
19	Type‐C	12	7	40.63 ± 13.9	28.24 ± 2.75	6	13	−0.86 ± 0.86	3	16
3	Type‐D	2	1	32 ± 2.45	27.13 ± 0.71	1	2	−0.7 ± 0.14	0	3
6	Type‐E	5	1	39.17 ± 12.13	28.13 ± 1.6	1	5	−0.85 ± 0.52	0	6
9	Type‐F	4	5	48.11 ± 16.06	29 ± 2.66	2	7	−1.6 ± 1.05	0	9
12	Type‐G	6	6	57.58 ± 5.19	28.2 ± 2.06	3	9	−1.95 ± 0.73	0	12
11	Type‐H	6	5	34.73 ± 13.8	27.35 ± 1.88	3	8	−0.59 ± 1.04	1	10
2	Type‐I	1	1	39.5 ± 1.5	27.01 ± 0.78	1	1	−0.5 ± 0.2	0	2

Written informed consent was obtained from all patients, and this study was approved and registered by the Ethics Committee of Cangzhou Central Hospital, IRB Approval Number: 2016‐053‐01.

### 2.6 Classified Cases

**FIGURE 2:**
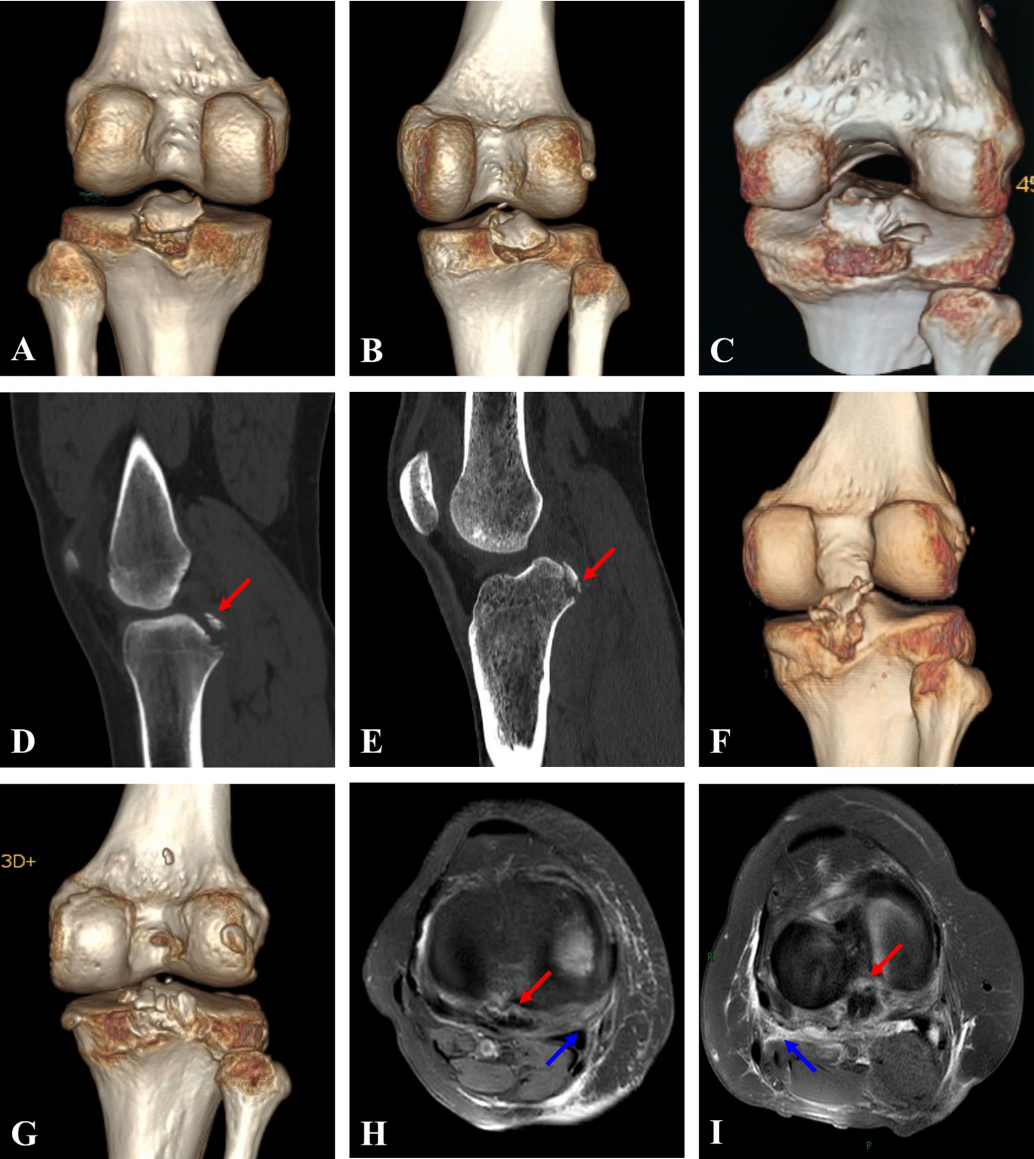
The CT (A:Type‐A, B:Type‐B, C:Type‐C, D:Type‐D, E:Type‐E, F:Type‐F, G:Type‐G) and the MRI (H:Type‐H, I:Type‐I) demonstrated that the types of posterior cruciate ligament avulsion fracture (red arrow) and the condition of soft tissue injuries (blue arrow).

Among 100 patients, nine representative cases of PCLTAF were selected based on the Xu‐Chen concise classification. These cases were presented using imaging data from the most optimal perspectives, and their treatment outcomes were reported. Among them, there were six male and three female patients; six cases were caused by sports injuries, and three cases resulted from traffic accidents. The average age was 45 years (range: 25–72 years). All patients underwent radiological evaluation, including X‐ray, CT, and MRI, to assess the fracture location and severity (Figure 2).

### 2.7 Treatment Strategy Based on Xu‐Chen Concise Classification

To achieve efficient integration between diagnostic classification and therapeutic decision‐making, this study developed a corresponding treatment decision model based on the Xu‐Chen concise classification System. In this model, fracture type is incorporated as a core variable within the clinical decision‐making pathway, allowing classification results to directly inform treatment strategies and enabling a closed‐loop transition from diagnosis to intervention.

For each fracture subtype, the model defines a standardized treatment protocol, establishing a decision‐making framework centered on the “classification–treatment strategy” sequence, with strong clinical operability and broad applicability. Within the Xu‐Chen concise classification, non‐operative indications for each subtype are also clearly integrated into the treatment pathway: Types A–E: If the avulsed PCL fragment shows no significant displacement, the knee remains overall stable, and imaging suggests preserved ligament continuity, conservative treatment is preferred (including immobilization, early quadriceps strengthening, and scheduled imaging follow‐up). In cases of minor displacement with acceptable ligament tension, observation with close monitoring may be considered. Types F and G: Often associated with comminution at the ligament insertion site or discontinuity of the PCL structure, resulting in poor knee stability and low rates of healing with conservative treatment. In such cases, early surgical intervention is generally recommended. Types H and I: These represent complex multi‐structural injuries with a high degree of instability. Conservative management often leads to poor outcomes, and combined ligament reconstruction or reinforced suture fixation is usually required.

For subtypes requiring surgical intervention, the treatment protocol is further refined into four standardized bundle‐weaving fixation techniques: (A) Whole‐bundle fixation (B) Medial bundle braiding and fixation (C) Lateral bundle braiding and fixation (D) Bundle‐weaving whole‐bundle fixation. In clinical application, surgeons can rapidly identify the fracture type through preoperative imaging and accurately infer the appropriate suture fixation method, surgical approach, and implant selection, thereby significantly improving the precision and efficiency of individualized treatment. Additionally, this model provides a theoretical basis for the personalized design of fixation plates and the clinical integration of suture‐based fixation techniques.

The development of this treatment strategy fully considered the anatomical characteristics of each fracture subtype, including the location of the fragment, degree of displacement, bone morphology, presence of osteoporosis, and involvement of the posterior ligamentous complex of the knee. These key factors form the foundation of pathway design and support the selection of surgical techniques, fixation configurations, and the need for concurrent ligament repair.

To further enhance the model's clinical utility and practical value, the research team developed the “Xu‐Chen concise classification and bundle‐weaving fixation technique” Reference Table (see Table 2), a quick‐reference tool designed to assist clinicians in efficiently matching classification results with recommended treatments and surgical plans. This contributes to the establishment of a closed‐loop support system linking imaging‐based diagnosis to therapeutic decision‐making.

**TABLE 2:** Xu‐Chen concise classification and bundle‐weaving fixation technique.

Classification and abbreviation		Fixation method and description	
Type‐A	Monoblock type: This type of method is suitable for simple, intact fractures without comminution.	Whole‐bundle fixation	Using either whole‐bundle fixation alone or bundle‐weaving whole‐bundle fixation based on the condition of the ligamentous bone fragment, the intact fracture fragment is typically secured within the fixation range of the plate and screws, achieving fracture stability.
Type‐B	Medial dominant split type: The medial side of the main fracture fragment is split, with small fragments that are difficult to fix.	Bundle‐weaving partial‐bundle fixation	The bundle‐weaving partial‐bundle fixation technique is used to stabilize the medial comminuted fracture fragments, while the main fracture fragment is typically secured within the fixation range of the plate and screws, enhancing overall load‐bearing capacity and stability.
Type‐C	Lateral dominant split type: The lateral side of the main fracture fragment is split, with small fragments that are difficult to fix.		Treatment approach: Similar to Type B, but with reinforced weaving fixation for the lateral comminuted fracture fragments.
Type‐D	Proximal dominant split type: Small, difficult‐to‐fix bone fragments appear at the proximal end of the main fracture fragment.	Bundle‐weaving whole‐Bundle fixation	Through anterior–posterior whole‐bundle weaving, the ligament is integrated into a unified structure. The main fracture fragment is typically secured within the fixation range of the plate and screws, ensuring both localized reinforcement and overall stability.
Type‐E	Distal dominant split type: Small, difficult‐to‐fix bone fragments appear at the distal end of the main fracture fragment.		Treatment approach: Same as Type D.
Type‐F	Quadrant comminution type: The fracture site shows comminution, often with a main bone fragment. Both cortical and cancellous bone coexist, mostly observed in young patients or those with well‐developed and strong ligaments.		The bundle‐weaving whole‐bundle fixation technique is used, ensuring that after ligament weaving, the main fracture fragment is secured with plate and screw fixation. This approach integrates the local structure into a stable unit, providing sufficient stability.
Type‐G	Complete footprint comminution type: The fracture region exhibits severe comminution, mostly consisting of thin cortical bone fragments. It is commonly seen in elderly or osteoporotic patients with thinner cortical bone.		For this type of injury, conventional fixation devices often fail to achieve stable fixation after ligament weaving. The novel fixation system with tendon‐weaving holes (Patent No.: ZL201620025670.3) enables bundle‐weaving whole‐bundle fixation, providing compression and stabilization for the entire fracture site, thereby enhancing overall stability.
Type‐H	Fracture with injury of medial ligament complex type: The fracture type is usually indeterminate.	Fracture fixation with ligament repair	Ligament‐weaving fracture fixation, combined with medial ligament complex repair, is performed to restore knee joint stability and function.
Type‐I	Fracture with injury of lateral ligament complex type: The fracture type is usually indeterminate.		Ligament‐weaving fracture fixation, combined with lateral ligament complex repair.

*Note:* All the above types are treated using the novel fixation system with tendon‐weaving holes (Patent No.: ZL201620025670.3) to achieve bundle‐weaving fixation.

**FIGURE 3:**
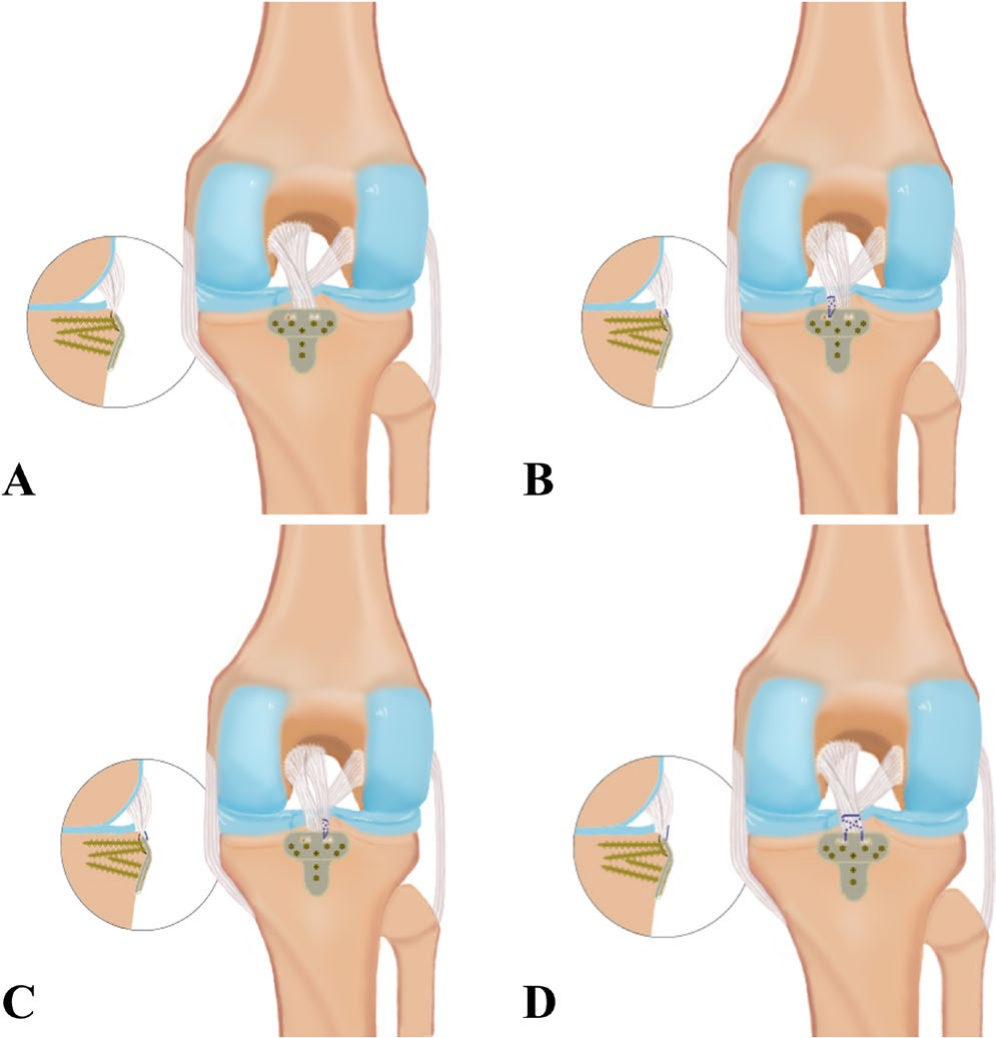
(A) Whole‐bundle fixation. (B) Medial bundle braiding and fixation. (C) Lateral bundle braiding and fixation. (D) Bundle‐weaving whole‐bundle fixation.

During surgery, the novel fixation system with tendon‐weaving holes (Patent No.: ZL201620025670.3) was applied, utilizing the Xu‐Chen concise classification for bundle‐weaving fixation. This approach is intuitive, simple, and highly practical, providing a comprehensive and reliable basis for clinical diagnosis and treatment decisions (Figure 3).

### 2.8 Anatomic Data Measurement

CT‐based 3D reconstruction was performed to determine the precise location and morphology of the fracture, while MRI imaging was used to assess soft tissue injuries, particularly in the PCL tibial attachment area. A 3D reconstruction technique was employed to create a knee joint bone structure model, accurately identifying the PCLTAF site.

During the reconstruction process, anatomic data such as the fracture site diameter, fragment size, shape, height, and width were measured. Additionally, the condition of the ligaments and soft tissues surrounding the knee joint was evaluated. A mirrored 3D‐printed PCLTAF fixation device of the contralateral limb was used for comparison. Combined with 3D‐printed surgical guides, this approach helped match appropriate internal fixation devices and surgical plans, effectively assisting in ligament reinforcement and fracture fixation.

In osteoporotic patients, traditional fixation methods are prone to failure due to factors such as age, bone quality, and comminution of fracture fragments. Special attention should be given to anatomic data in these patients when developing surgical plans to provide optimal clinical support.

### 2.9 Surgical Technique and Internal Fixation Device Design and Application

All patients underwent combined spinal‐epidural anesthesia or femoral nerve block anesthesia in the prone position. Surgery was performed on the affected limb under a pneumatic tourniquet. The skin was routinely disinfected with complex iodine, and a sterile drape was applied. The incision was designed as a posteromedial transverse arcuate incision, extending from the posterior midline of the knee joint obliquely downward and inward along the medial head of the gastrocnemius muscle. The skin, subcutaneous tissue, and deep fascia were incised, and blunt dissection was performed between the semitendinosus muscle and the medial head of the gastrocnemius muscle to expose the posterior tibial plateau and fracture site.

**FIGURE 4:**
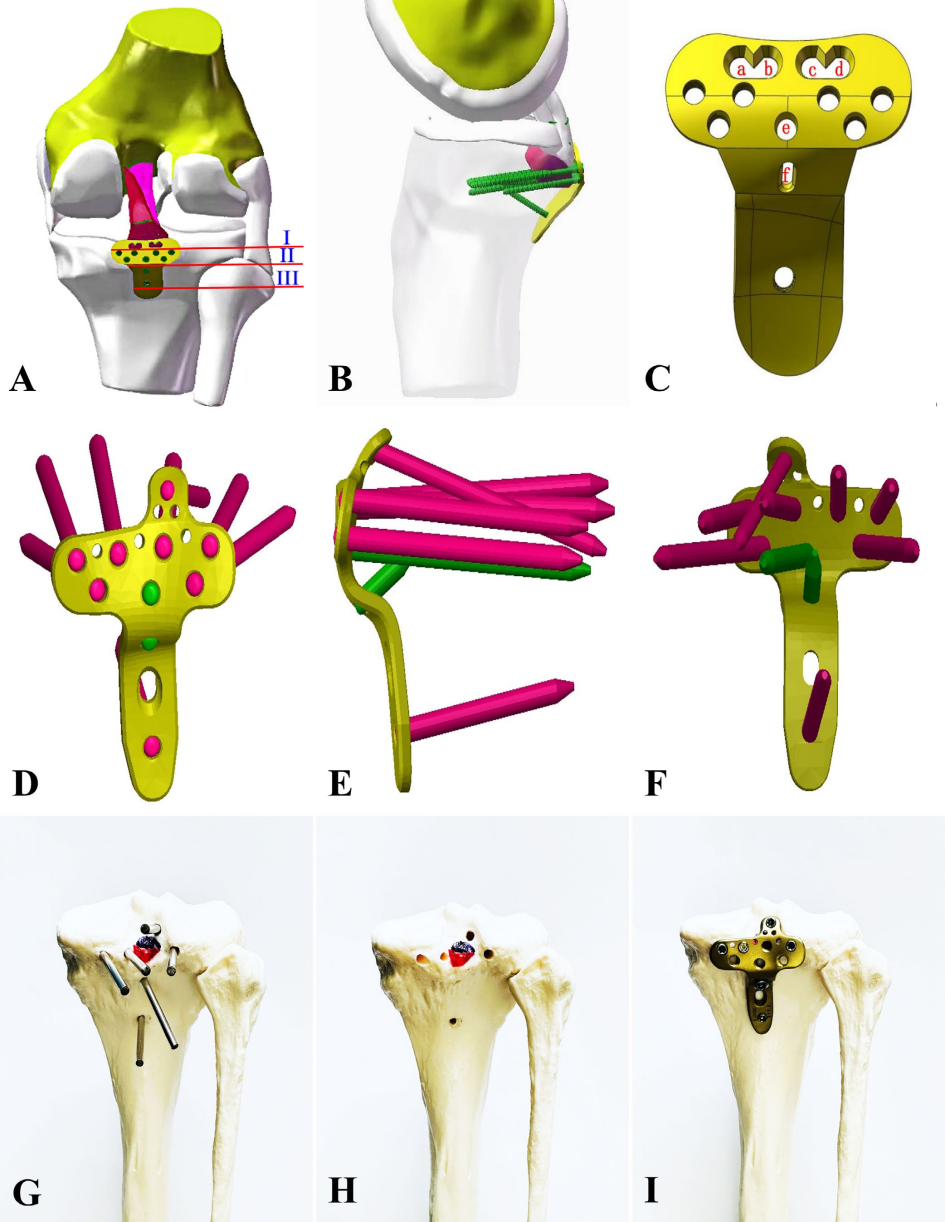
(A–C) Schematic illustration of the three‐zone bundled weaving fixation principle. Semi‐threaded holes (a–d) serve as fixation points for screws and ligament weaving, while optional compression screw holes (e, f) enhance lateral plate compression and overall conformity. The fixation system is divided into three functional zones: Zone I (tendon weaving region), Zone II (bone block‐plate fixation region), and Zone III (fixation stability region). (D–F) Model diagrams of the Third‐generation fixation device (Only the TC‐R type is shown, while the TC‐L type is also available, with designs distinguishing between left and right knee joint applications), featuring enhancements such as the protruding structure at the top of the transverse plate surface, bilateral anatomical design of the lateral structure, and breakpoint design on the dorsal side. (G–I) Application scenarios of the commercialized product in the fixation model.

**FIGURE 5:**
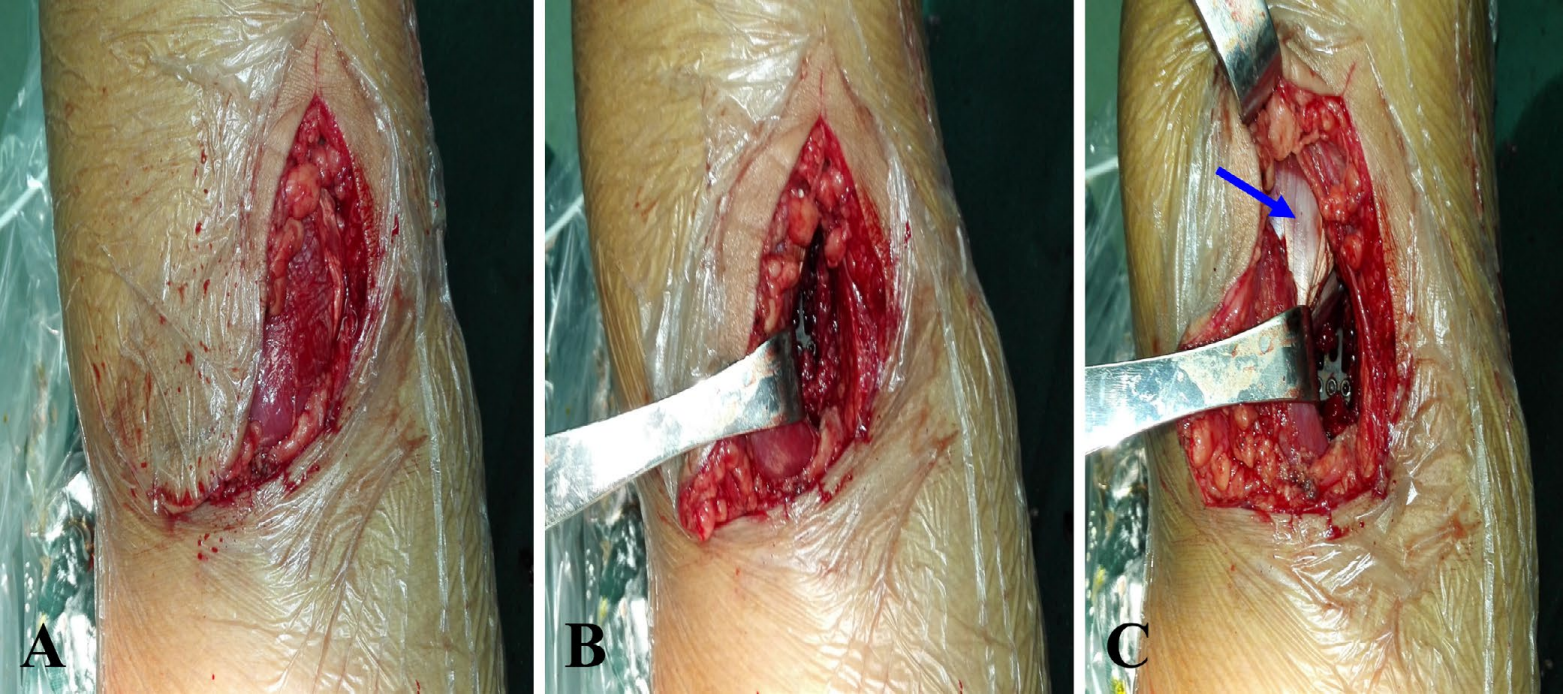
Intraoperative exposure and fixation procedure illustration. (A) Posterior inverted L‐shaped incision of the knee to expose the surgical field. (B) At the tibial insertion of the PCL, a bundle‐ weaving fixation technique is selected based on the Xu‐Chen concise classification. A tendon‐weaving‐hole fixation device with mirrored design (TC‐L or TC‐R) is applied according to the affected limb for directional compression and fixation. (C) Surgical approach through the intermuscular plane along the medial border of the gastrocnemius (blue arrow), clearly demonstrating the anatomical relationship between the gastrocnemius and the fixation device while effectively avoiding the Popliteal artery, vein, and tibial nerve.

**FIGURE 6:**
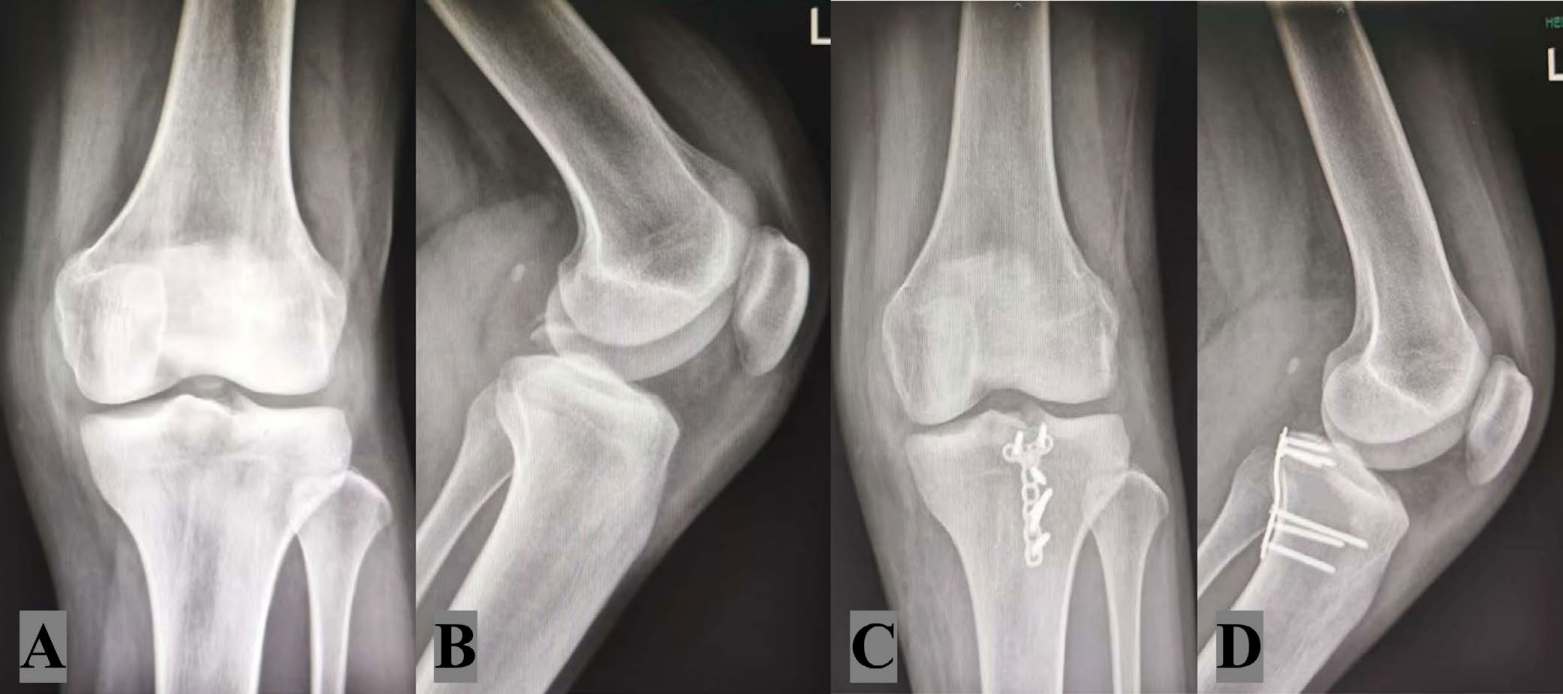
Preoperative (A, B) and postoperative (C, D) X‐ray images of the knee joint. A novel fixation system with tendon‐weaving holes was used, combining open reduction with a classified bundle‐weaving repair technique for injury fixation. Postoperative images show internal fixation materials (plates, screws, and woven sutures), providing stable support to promote fracture healing and functional recovery.

**FIGURE 7:**
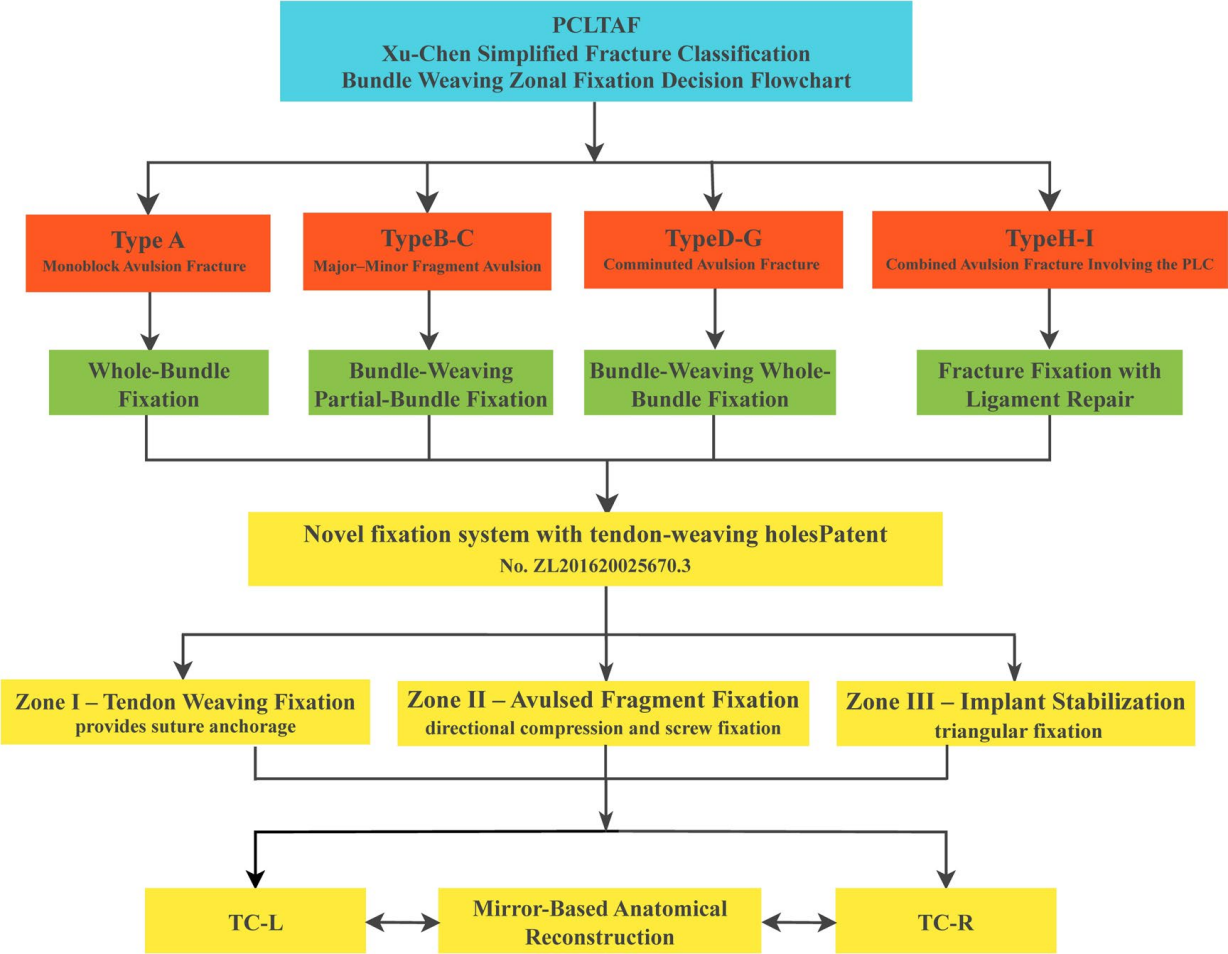
PCLTAF:Xu‐Chen concise classification—bundle weaving—zonal fixation decision flowchart.

The surgical procedure was established based on the bundle‐weaving fixation strategy and the “Three‐zone fixation model” generated by the novel fixation system with tendon‐weaving holes: Zone I: Tendon‐weaving area; Zone II: Bone fragment plate fixation area; Zone III: Fixation device stabilization area (Figure 4). This treatment strategy quantifies surgical techniques in clinical practice (see Table 3). The surgical steps are as follows: In the tibial anterior drawer position, high‐strength sutures were used to reinforce avulsed or severely damaged ligaments with bundle‐weaving fixation according to the established classification method, ensuring sufficient suture tail length. After fracture reduction, the PCL tibial avulsion fragment was temporarily fixed. The novel anatomical internal fixation system with tendon‐weaving holes (various modified models have been granted national utility model patents, Patent No.: ZL201620025670.3) was placed at the posterior midline of the tibial plateau. The woven sutures were passed anteriorly to posteriorly through the tendon‐weaving holes in the fixation system. A point‐contact anti‐slip fixation system was used to compress the comminuted bone fragments, and compression screws and locking screws were applied, ensuring that the screw direction did not exceed the tibial plateau. Intraoperative fluoroscopy confirmed the satisfactory positioning of the fixation system with tendon‐weaving holes. The knee joint was flexed and extended to assess stability, and finally, the incision was closed. This comprehensive repair strategy provides strong fixation, enhances the stability of bone fragments and ligaments, and optimally supports knee joint function recovery. Intraoperative exposure and postoperative radiographic findings (Figures 5 and 6). PCLTAF:Xu‐Chen concise classification—bundle weaving—zonal fixation decision flowchart (Figure 7).

**TABLE 3:** Zoning fixation strategy of the fixation system with tendon‐weaving holes.

Zonal division	Braided fixation steps
Zone I	Woven Tendon Zone, During the repair process, the damaged ligament and bone fragment are exposed to facilitate precise reconstruction: First, the surgical field is exposed to reveal the tibial attachment area of the posterior cruciate ligament. Then, woven suturing is performed using a No. 2 suture (diameter: 0.5–0.599; Poisson's ratio: 0.42; elastic modulus: 1100–1600 cN/dtex; maximum load: ≥ 34.5 N) in a figure‐eight pattern. The needle exits on the dorsal side of the ligament, generating a downward and anterior force. The suture tail passes through the nail hole in Fixation Device Zone I, is tightened and knotted or secured with a clip, restoring the initial anatomical position of the ligament insertion. Two weaving techniques are utilized: bundle‐separated weaving with bundle‐separated fixation and whole‐bundle weaving with whole‐bundle fixation. These techniques are applicable to Type‐A, Type‐B, Type‐C, Type‐D, Type‐E, Type‐F, and Type‐G cases, ensuring the ligament avoids re‐tearing during healing, enhancing tensile strength, and improving long‐term stability.
Zone II	Bone Fragment Plate Fixation Zone, after tendon weaving, the avulsed bone fragment is stabilized to ensure structural integrity: First, the bone fragment is repositioned to its original anatomical location, ensuring a precise fit and optimal contact with surrounding structures. Following reduction, bone plate fixation is performed. A customized bone plate is selected to overlay and secure the fragment, effectively preventing displacement. For cases classified as Complete Footprint Comminution Type and certain Quadrant Comminution Type, where the bone fragment is severely comminuted and dispersed, traditional screw fixation in Main Bone Fragment Zone II is not feasible. In such cases, an alternative fixation method is employed. This involves whole‐bundle weaving at the distal tendon, encircling the tendon‐bone interface. After traction‐assisted reduction, the suture tail is passed through the tendon‐weaving hole fixation system to secure the structure. Additionally, the transverse plate compression effect further reinforces stability, ensuring long‐term fixation of the fragment.
Zone III	Fixation Device Stabilization Zone, to enhance the overall adherence and stability of the fixation device and prevent displacement or failure, the following steps are implemented: Preoperative planning is crucial in this process. First, appropriate compression screws and locking screws are selected for fixation. In cases of severe comminuted fractures or concurrent osteoporosis, achieving adequate fixation strength is essential. This requires a comprehensive assessment of screw type, length, and diameter to ensure optimal stability. Additionally, the screw placement must allow for both bone fragment fixation via the bone plate and secure anchoring within the tibial stabilization zone to maximize structural integrity.

*Note:* The “Three‐Zone Method” classification‐based bundle‐weaving fixation technique integrates multiple key technologies, including woven tendon repair, bone fragment plate fixation, and fixation device stabilization, to achieve comprehensive structural reconstruction and enhanced biomechanical stability.

### 2.10 Postoperative Management

All patients undergoing PCLTAF repair follow a standardized postoperative management protocol to ensure optimal recovery. In the early postoperative phase, movement is restricted, and cold compression and limb elevation are applied to reduce swelling and pain. The knee is immobilized in 20° flexion using a hinged brace to prevent excessive flexion and extension. Regular X‐ray evaluations are performed to monitor fracture healing, and MRI scans are conducted when necessary to assess ligament integrity and fracture site recovery in detail. On postoperative day 3, after drain removal, passive full‐range motion is initiated 2–3 times daily. By week 2, protected active knee exercises begin to promote joint mobility and muscle strength recovery. Complications such as deep vein thrombosis, knee stiffness, infection, postoperative swelling, and hematoma are meticulously documented.

Follow‐up was conducted at predefined time points, specifically at 3, 6, 12, 18, and 24 months postoperatively through outpatient visits. Each follow‐up included a systematic physical examination, with particular emphasis on the posterior drawer test. Functional evaluation was performed using the Lysholm Knee Score and the Tegner Activity Scale. Radiographic or MRI reassessment was performed as needed based on the patient's recovery status. During follow‐up, rehabilitation progress, occurrence of complications, and functional outcomes were documented. To ensure continuity of follow‐up, telephone interviews were conducted in addition to in‐person visits.

### 2.11 Surgical and Clinical Outcomes

This study utilized a novel fixation system with tendon‐weaving holes to design and reconstruct a repair system for PCLTAF. All patients underwent fracture reconstruction using a 3D‐printed guide plate.

The mean operative time was 77.2 min (range: 60–95 min), and the mean blood loss was 23.3 mL (range: 15–40 mL). The average follow‐up period was 17.89 months (range: 12–22 months).

A total of nine patients were diagnosed with PCLTAF and underwent fixation using the Novel fixation system with tendon‐weaving holes, without additional fractures in other locations. (Table 4).

**TABLE 4:** Novel tendon‐weaving fixation treatment for 9 PCLTAF cases of Xu‐Chen concise classification subtypes.

Patient number	Sex	Classification	Raiding and fixation	OT (min)	BL (mL)	Preoperative lysholm score	Postoperative lysholm score	Preoperative tegner score	Postoperative tegner score	Complications	Follow‐up (months)
1	F	Type‐A	Whole‐bundle fixation	60	15	28	98	4	9.6		15
2	M	Type‐B	Bundle‐weaving partial‐bundle fixation	70	20	29	96	3	9.8		16
3	F	Type‐C		65	20	26	98	3	9.5		14
4	M	Type‐D		75	15	28	97	2	9.7		12
5	M	Type‐E	Bundle‐weaving whole‐bundle fixation	80	20	27	98	3	9.8		18
6	M	Type‐F		75	25	29	97	2	9.6		26
7	F	Type‐G		85	20	24	96	4	9.3	Incision necrosis	22
8	M	Type‐F Type‐H	Bundle‐weaving whole‐bundle fixation with ligament repair	95	40	24	98	3	9.5		18
9	M	Type‐A Type‐I	Whole‐bundle fixation with ligament repair	90	30	26	97	2	9.3		20
Mean		—	—	77.2	23.3	26.78	97.22	2.89	9.56	—	17.89

Abbreviations: BL, blood loss; OT, operation time.

At the final follow‐up, all patients had resumed normal activities or high‐intensity exercise without the need for assistive devices. The mean Lysholm score significantly improved from 26.78 preoperatively to 97.22 postoperatively, and the mean Tegner activity level increased from 2.89 to 9.56. No complications such as deep vein thrombosis, knee stiffness, postoperative swelling, hematoma, or infection were observed during the follow‐up period. Only one elderly patient developed superficial wound necrosis, which healed after repeated dressing changes. No cases of fixation failure, joint instability, or secondary fractures were identified. Imaging showed no signs of radiolucent lines at the bone–implant interface, implant displacement, or prosthesis failure, indicating satisfactory bone healing.

## 3 Discussion

This study is the first to propose the “Xu‐Chen concise classification” for PCLTAFs. Based on this classification, we developed a corresponding bundle‐weaving fixation technique and designed a novel fixation system with tendon‐weaving holes. Through surgical application in nine patients with PCLTAF, we systematically demonstrated the fixation strategies tailored to each subtype and their respective clinical outcomes. Notably, the technique achieved stable fixation even in cases involving comminuted fractures or osteoporotic bone, indicating strong clinical relevance. This approach provides a novel and systematic pathway for the treatment of PCLTAF.

### 3.1 Xu‐Chen Concise Classification

PCLTAF often presents on plain radiographs as small bone fragments near the ligament attachment site. Occasionally, larger bone fragments may be observed, typically projected within the joint space. These features can easily be misinterpreted as intra‐articular loose bodies. Therefore, accurate preoperative identification is crucial for specific and reliable diagnosis [16]. In 1959, Meyers et al. [17] proposed a rudimentary three‐type classification of PCLAF based on plain radiographs. However, this system does not adequately capture the morphological characteristics of avulsion fractures and offers limited clinical guidance [18]. Building upon the Meyers classification, Ren et al. [19] further subdivided PCLTAF into multiple subtypes based on CT imaging, considering displacement, extent of involvement, and fragment integrity. Nonetheless, this system is relatively complex, making it difficult to apply in emergency settings or intraoperatively. Moreover, it lacks integration with MRI evaluation of commonly associated posterior ligamentous complex injuries, overlooking key posterior soft tissue structures. As such, this classification emphasizes form over function and provides limited clinical utility. Based on the analysis of 100 cases of PCLTAF, this study—integrating CT and MRI imaging and employing a manual classification approach—proposes for the first time the Xu‐Chen concise classification system. Centered on the tibial insertion of the PCL, this system categorizes avulsion fractures into nine spatial types according to anatomical orientation. It comprehensively considers the location, morphology, degree of comminution of the fracture fragments, and the status of the posterior ligamentous complex. The classification aims to provide an intuitive and standardized framework for describing the spatial characteristics and extent of fracture involvement based on CT and MRI findings. In this study, Type‐A (Monoblock Type) was the most common in clinical practice, accounting for 30% of cases, with a mean patient age of 37.37 ± 10.84 years. In contrast, Type‐F (Quadrant Comminution Type) and Type‐G (Complete Footprint Comminution Type) were associated with older patient populations, with mean ages of 48.11 ± 16.06 years and 57.58 ± 5.19 years, respectively. The comminuted nature of these fracture types may be related to age‐associated declines in bone strength. The bone mineral density (BMD) T‐scores for these two types were −1.6 ± 1.05 and −1.95 ± 0.73, respectively—the lowest among all types—suggesting a significant reduction in bone mass. The second most common type was Type‐C (Lateral Dominant Split Type) comprising 19% of cases. This subtype typically presents with lateral bone fragments, which may be due to their location at the outermost edge of the ligament footprint, making them more susceptible to traction or shear forces during injury. Type‐I (Fracture with Lateral Ligament Complex Type) accounted for only 2%, making it the rarest subtype. This pattern is likely related to high‐energy trauma causing excessive varus stress and external tibial rotation of the knee. Schulz et al. [20] reported that of 494 patients, the most common causes of injury were traffic accidents (45%) and sports‐related injuries (40%), including motorcycle accidents (28%) and soccer‐related injuries (25%). Schlumberger et al. [21] reported that sporting activity was the leading cause of PCL lesions, accounting for 388 patients (38.8%), followed by traffic accidents in 350 patients (35.0%). In this study, sports‐related injuries were the leading cause of PCLTAF, accounting for 72 cases, followed by traffic‐related injuries with 28 cases, yielding a ratio of 2.6:1. The Xu‐Chen concise classification provides systematic guidance for the diagnosis and treatment of PCLTAF by enabling precise localization of fracture fragments and emphasizing the identification and assessment of key posterior soft tissue injuries of the knee. This classification system facilitates rapid recognition of fracture types requiring surgical intervention and addresses the limitations of traditional classifications, such as unclear decision‐making pathways and insufficient clinical guidance.

### 3.2 Bundle‐Weaving Fixation Technique

White et al. [16] Injury to the PCL of the knee most commonly involves a tear of the collagenous fibers of the ligament. PCL bundle‐weaving reinforcement reconstruction has gained increasing clinical attention, with the key objective of maintaining ligament tension postoperatively to prevent posterior tibial translation resulting from PCL insufficiency [22, 23, 24, 25]. Based on the Xu‐Chen concise classification, this study is the first to propose a classification‐based conservative treatment strategy and innovatively design four PCL tibial footprint–oriented bundle‐weaving reconstruction techniques: (1) Whole‐bundle fixation, (2) Medial bundle braiding and fixation, (3) Lateral bundle braiding and fixation, (4) Bundle‐weaving whole‐bundle fixation. This framework establishes a classification‐guided, bundle‐specific treatment algorithm that enables precise matching between fracture subtype and surgical pathway. In clinical practice, patients routinely undergo X‐ray, CT, and MRI to comprehensively assess fracture location, morphology, comminution characteristics, involvement of the posterior ligamentous complex, and degree of osteoporosis. Based on this evaluation, the suitability for conservative management or a corresponding surgical strategy is determined using the Xu‐Chen concise classification and bundle‐weaving fixation technique scale. For Type‐A (Monoblock Avulsion Fracture), using either whole‐bundle fixation alone or bundle‐weaving whole‐bundle fixation based on the condition of the ligamentous bone fragment, an “figure‐of‐8” bundle‐weaving whole‐bundle fixation is applied at the tibial insertion of the PCL. Sutures are tied in an anterograde fashion through tendon‐weaving anchor holes located above the transverse plate of the novel fixation system with tendon‐weaving holes, reconstructing the local anatomic footprint. For Types B, C, D, and E (collectively termed Major–Minor Fragment Avulsion), the anatomical orientation of the major fragment is first identified. A localized bundle‐weaving repair or whole‐bundle reconstruction is then selected based on the location of the torn bundle zone, enabling functional reattachment and anatomical restoration. For Types F and G (Comminuted Avulsion Fractures), bundle‐weaving whole‐bundle fixation is performed at the PCL tibial insertion zone to reconstruct the entire ligament footprint. For Types H and I (Combined Avulsion Fractures involving the posterior ligamentous complex), preoperative MRI is essential to assess the extent of posterior ligamentous complex injury and to determine the need for concurrent repair. PCL reconstruction strategies follow the same principles as Types A–G, allowing targeted and individualized treatment planning. The bundle‐weaving fixation technique reference scale is concise, practical, and easy to implement. Built upon the Xu‐Chen concise classification, it provides a standardized decision‐making pathway for the treatment of PCLTAF and supports surgeons in developing more precise, individualized strategies in complex cases.

### 3.3 Design and Application Features of the Novel Fixation System With Tendon‐Weaving Holes

In the treatment of PCLTAF, repairing the PCL substance is equally important as addressing the tibial avulsion fracture. Endobutton fixation, as a type of elastic fixation method [26], carries a risk of fragment fragmentation during drilling, especially for thin and small bony fragments [27]. Moreover, when the fixation device traverses the tibial tunnel, it often induces a horizontal “windshield wiper effect” and a vertical “bungee effect” [28]. Several studies have reported a relatively high risk of postoperative knee joint fibrosis following arthroscopic treatment of PCL‐related injuries [18]. Posterior interval puncture procedures may compromise the blood supply to the fracture site, which is unfavorable for bone healing [29]. Intraoperatively, it is difficult to completely avoid iatrogenic damage to the articular cartilage. To obtain an adequate surgical field, the posterior interval synovium often needs to be removed, which further increases surgical trauma [30]. Clinical experience has shown that accurate reduction is often difficult to achieve with Endobutton fixation. The fixation force is predominantly loaded on the ligamentous tissue adjacent to the PCL tibial insertion rather than on the bone fragment itself, which may result in difficulty in achieving anatomical reduction or even fragment flipping, thereby compromising fixation stability. In a study on athlete injuries, Borque et al. [5] reported that the mean return‐to‐play (RTP) time was significantly longer in athletes with PCL‐based injuries compared to those with ACL‐based injuries (15.2 vs. 11.9 months, *p* < 0.01). Since 2016, based on large‐scale anatomical data analysis of the posterior central region of the tibial plateau, this study has developed a mini “T”‐shaped anatomical fixation plate specifically designed for the posterior aspect of the knee joint to address the characteristics of PCLTAF. A national patent was filed for the first‐generation device, which innovatively solved the intraoperative challenge of achieving precise conformity between the fixation plate and the inclined, irregular surface of the posterior tibial plateau. Although the first‐generation device demonstrated favorable clinical outcomes, intraoperative findings revealed that the number of tendon‐weaving anchor holes on the top surface of the transverse plate was limited, making it difficult to achieve effective longitudinal reinforcement aligned with the orientation of ligament fibers. To address this issue, a second‐generation device was developed with an optimized design featuring a single row of four semi‐threaded, gourd‐shaped holes arranged in interconnected pairs. This design preserved the functionality of suture‐weaving channels while allowing for screw insertion, thereby significantly improving the feasibility of fixation in proximal comminuted fractures. Surgeons can flexibly employ a combined fixation strategy using both sutures and screws according to the fracture type and bone quality, enhancing reduction accuracy and fixation stability. Based on the Xu‐Chen concise classification, the third‐generation device was further refined to align with the corresponding bundle‐weaving techniques. The reinforced PCL can be anchored onto the transverse plate via the Adjustable Semi‐threaded Ligament Anchor Hole, effectively overcoming the instability associated with traditional elastic fixation methods.

Lukas Willinger et al. [31] reported that small bone fragments are prone to refracture during screw fixation. However, in actual clinical practice, even relatively large and intact fragments carry a risk of splitting during screw insertion. In addition, a single screw provides limited anti‐rotational stability, while multiple screws are often restricted by the fragment's size and spatial orientation, making stable fixation difficult to achieve. Although suture anchors can be used to fix woven ligaments to comminuted fracture zones, their effectiveness depends heavily on adequate bone quality and may be compromised by loosening, pull‐out, or insufficient fixation strength [32]. The third‐generation novel fixation system with tendon‐weaving holes developed in this study incorporates a novel “protrusion” structure with break‐point design on the upper surface of the transverse plate. This structure contains multi‐axial screw holes and tendon‐weaving anchor holes. Using 2 mm multi‐directional screws combined with directed compressive force from the fixation plate, the device enables flexible yet stable fixation of small and thin avulsion fragments. It also avoids the fibrous projection zone of the PCL tibial footprint, thereby reducing the risk of iatrogenic injury during surgery while providing essential local stability. Moreover, the tendon‐weaving anchor holes offer secure suture attachment points for ligament tears near the tibial insertion, especially in cases with osteoporosis or severely comminuted avulsion fractures. These anchor points serve as stable supports in enhanced bundle‐weaving reconstruction, demonstrating the system's multidimensional adaptability. In addition, to account for the anatomical differences between the medial and lateral posterior margins of the tibial plateau, mirrored modeling technology was used to design two anatomically contoured fixation plates: a right‐sided version (TC‐R) and a left‐sided version (TC‐L), thereby improving anatomical compatibility and surgical fit.

With the rapid advancement of 3D printing technology, the concept of personalized treatment has been widely validated in knee surgery [33, 34]. In this study, for the first time, 3D‐printed guiding templates were applied in the treatment of PCLTAF using the novel fixation system with tendon‐weaving holes based on patient‐specific imaging data. To accommodate individual variations in the yy curvature of the tibial plateau, the guide was optimized to improve conformity with the bone surface, thereby enhancing the directional compressive capability on fracture fragments. Particularly in the ligament attachment area, the guide allows for pre‐setting the orientation of screw trajectories, which minimizes the risk of ligament injury and reduces the likelihood of screw penetration into the articular surface. For patients with osteoporosis or poor bone quality, screw length and insertion angle can be fine‐tuned based on bone mineral density, further increasing pullout resistance and better fulfilling the clinical demands of precision treatment [35].

### 3.4 Clinical Validation and Data Analysis

A total of nine patients with PCLTAF were included in this study, with a mean follow‐up duration of 17.89 months. All nine subtypes defined in the Xu‐Chen concise classification were represented. All patients underwent surgery via a posteromedial inverted “L”‐shaped incision. Guided by the bundle‐weaving strategy based on the Xu‐Chen concise classification, each case was treated using the Novel fixation system with tendon‐weaving holes, and favorable postoperative outcomes were achieved. In previous studies on open reduction and internal fixation (ORIF) for PCLTAF, a systematic review and meta‐analysis [18] showed that, in acutely treated knees across three studies, the mean Lysholm score improved from 35.4 preoperatively to 94.8 postoperatively. Singla et al. [36] demonstrated that the mean Lysholm score improved from 82 preoperatively to 92 postoperatively. Seitz et al. [37] showed that all 26 patients had a pre‐injury Tegner score ≥ 6, and 23 of them maintained a postoperative score of ≥ 6. In the present study, the mean preoperative Lysholm score was 26.78, which significantly improved to 97.22 postoperatively. The Tegner score increased from 2.89 to 9.56, indicating marked improvements in knee joint function and physical activity level compared to preoperative status. These results demonstrate effective restoration of knee stability under the applied treatment protocol. The variation in outcome scores may be attributable to several factors: (1) Differences in initial injury types—for instance, Type‐H and Type‐I often involve concomitant injuries to the posterior ligamentous complex, requiring combined repair; (2) Variability in pre‐injury activity levels and postoperative rehabilitation compliance; (3) Differences in patient age distribution.

This study established an integrated treatment framework for PCLTAF, encompassing injury classification, surgical strategy, and implant design. The Xu‐Chen concise classification, grounded in fracture mechanism and anatomical characteristics, provides a standardized and practical basis for preoperative planning and intraoperative decision‐making. Based on this system, a reconstruction approach combining bundle‐weaving techniques with a novel fixation system with tendon‐weaving holes was developed to enable precise matching between fracture subtype and fixation strategy, thereby enhancing biomechanical strength and stability. The newly designed implant incorporates anatomical protrusions, adjustable screw trajectories, and 3D‐printed surgical guide compatibility, significantly improving early fixation outcomes and addressing limitations of traditional devices. Preliminary clinical results suggest favorable stability, complication control, and functional recovery, with good adaptability in complex cases such as comminuted avulsions and osteoporotic bone. Collectively, this study proposes a reproducible and scalable closed‐loop solution for the precise and standardized management of complex PCLTAF.

This study has certain limitations in the early stages of exploring new technologies. Due to the small sample size of the surgical cases using the novel bifurcated woven fixation system, its widespread applicability still needs to be validated through larger‐scale multicenter studies. The short follow‐up period also prevented the assessment of long‐term knee joint function recovery. The experience and technical proficiency of the surgeons may affect the surgical outcomes, and this study did not fully eliminate the impact of the learning curve. Moreover, the cost of the tendon‐woven internal fixation system primarily comes from the special sutures, bone plates, fixation screws, and the design and production of personalized 3D‐printed guides, which may increase healthcare expenses. However, the use of 3D‐printed guides during surgery can improve the accuracy of fracture reduction, reduce adjustment time, and shorten the operation duration, thereby lowering operating room costs and anesthesia fees. Additionally, the woven fixation technique enhances the stability of ligament repair, potentially reducing the risk of postoperative displacement and revision surgeries, decreasing long‐term healthcare costs, and improving patient quality of life and socioeconomic benefits. Furthermore, as this study is a retrospective analysis, it lacks a prospective comparison between the new and traditional fixation methods, making it impossible to directly quantify the advantages and disadvantages of the two. It also did not account for potential influences from differences in healthcare resources and surgeon training levels. Future research should increase the sample size, extend the follow‐up period, and conduct prospective studies to more comprehensively and objectively evaluate the clinical value of this classification system and fixation technique.

## 4 Prospects of Clinical Application

This study established a closed‐loop reconstruction system based on the “Xu‐Chen concise classification–bundle weaving–zonal fixation with tendon‐weaving hole implant,” demonstrating promising clinical applicability. The proposed classification system features a clear structure and is easy to apply, making it suitable for adoption across various levels of medical institutions to achieve rapid identification and standardized preoperative planning for PCLTAF. The matched bundle‐weaving fixation techniques, combined with zonal fixation using the tendon‐weaving hole implant, offer a novel approach for the precise treatment of complex fracture types. However, challenges remain in the clinical translation of this system, such as inter‐surgeon variability in understanding the classification and the need for individualized implant adaptation, which may require the aid of 3D‐printed surgical guides.

Future work will focus on the following directions: (1) Conducting multicenter clinical cohort studies to evaluate the applicability of the classification–bundle weaving system across diverse patient populations and stages of injury; (2) Optimizing the implant design to improve intraoperative usability and adaptability; (3) Exploring the integration of digital navigation technologies to further enhance the precision and intelligence of PCLTAF treatment.

## 5 Conclusions

PCLTAF is a complex injury that significantly impacts knee joint function. The novel Xu‐Chen concise classification introduces bundle‐weaving fixation, integrating precise classification, and personalized fixation strategies with 3D‐printed surgical guide technology. This approach enables accurate localization and fixation of fracture fragments, restoring knee stability and function, optimizing the treatment process, and providing a new direction for managing complex fractures.

## Author Contributions

G.C. and W.X. designed the study. Y.C. performed data analysis, model development, and visualization. W.W. and C.Y. were responsible for data collection and organization. G.C. drafted the manuscript, while W.X. provided revisions and suggestions. All authors contributed to the editing and revision of the manuscript, and all have read and approved the final version.

## Ethics Statement

This study was approved by the Ethics Committee of Cangzhou Central Hospital (2016–053‐01). All patients provided informed consent and signed a written consent form before participation in the study.

## Conflicts of Interest

The authors declare no conflicts of interest.

## Data Availability

Research data are not shared.

## References

[1] B. Cengiz and S. Karaoglu , “Case Report of Concomitant Avulsion Fractures of the Medial Meniscus and Posterior Cruciate Ligament,” Medicine 100, no. 50 (2021): e28273. 34918701 10.1097/MD.0000000000028273 PMC8677961

[2] C. Gwinner , S. Kopf , A. Hoburg , N. P. Haas , and T. M. Jung , “Arthroscopic Treatment of Acute Tibial Avulsion Fracture of the Posterior Cruciate Ligament Using the TightRope Fixation Device,” Arthroscopy Techniques 3, no. 3 (2014): e377–e382. 25126507 10.1016/j.eats.2014.02.005 PMC4130139

[3] H. Guo , Y. Zhao , L. Gao , et al., “Treatment of Avulsion Fracture of Posterior Cruciate Ligament Tibial Insertion by Minimally Invasive Approach in Posterior Medial Knee,” Frontiers in Surgery 9 (2023): 885669. 36684149 10.3389/fsurg.2022.885669 PMC9852621

[4] W. M. Wind, Jr. , J. A. Bergfeld , and R. D. Parker , “Evaluation and Treatment of Posterior Cruciate Ligament Injuries: Revisited,” American Journal of Sports Medicine 32, no. 7 (2004): 1765–1775. 15494347 10.1177/0363546504270481

[5] K. A. Borque , M. Jones , G. Balendra , et al., “High Return to Play Rate Following Treatment of Multiple‐Ligament Knee Injuries in 136 Elite Athletes,” Knee Surgery, Sports Traumatology, Arthroscopy 30, no. 10 (2022): 3393–3401. 10.1007/s00167-022-06926-3 35279739

[6] M. H. Meyers and F. M. McKeever , “Fracture of the Intercondylar Eminence of the Tibia,” Journal of Bone and Joint Surgery (American Volume) 52, no. 8 (1970): 1677–1684. 5483091

[7] C. D. Harner and J. Höher , “Evaluation and Treatment of Posterior Cruciate Ligament Injuries,” American Journal of Sports Medicine 26, no. 3 (1998): 471–482. 9617416 10.1177/03635465980260032301

[8] G. C. Fanelli , B. F. Giannotti , and C. J. Edson , “Arthroscopically Assisted Combined Posterior Cruciate Ligament/Posterior Lateral Complex Reconstruction,” Arthroscopy: The Journal of Arthroscopic & Related Surgery 12, no. 5 (1996): 521–530. 8902124 10.1016/s0749-8063(96)90189-9

[9] E. M. Escobedo , W. J. Mills , and J. C. Hunter , “The “Reverse Segond” Fracture: Association With a Tear of the Posterior Cruciate Ligament and Medial Meniscus,” American Journal of Roentgenology 178, no. 4 (2002): 979–983. 11906886 10.2214/ajr.178.4.1780979

[10] A. A. Khalifa , M. E. Elsherif , E. Elsherif , and O. Refai , “Posterior Cruciate Ligament Tibial Insertion Avulsion, Management by Open Reduction and Internal Fixation Using Plate and Screws Through a Direct Posterior Approach,” Injury 52, no. 3 (2021): 594–601. 33023741 10.1016/j.injury.2020.09.058

[11] K. C. Lin and Y. W. Tarng , “A Strategy to Prevent Complications of Hyperextension Type Tibial Plateau Fracture,” European Journal of Orthopaedic Surgery & Traumatology 31 (2021): 71–78. 32715326 10.1007/s00590-020-02739-7

[12] H. Liu , J. Liu , Y. Wu , et al., “Outcomes of Tibial Avulsion Fracture of the Posterior Cruciate Ligament Treated With a Homemade Hook Plate,” Injury 52, no. 7 (2021): 1934–1938. 33934882 10.1016/j.injury.2021.04.042

[13] H. Qi , Y. Lu , M. Li , et al., “Open Reduction and Internal Fixation of the Tibial Avulsion Fracture of the Posterior Cruciate Ligament: Which Is Better, a Hollow Lag Screw Combined With a Gasket or a Homemade Hook Plate?,” BMC Musculoskeletal Disorders 23, no. 1 (2022): 143. 35148737 10.1186/s12891-022-05096-0 PMC8840316

[14] P. Kumar , S. Aggarwal , G. Gupta , S. Sharma , A. Dadra , and V. Goni , “Concurrent Avulsion of Posterior Cruciate Ligament and Semimembranosus: A Case‐Based Discussion and Literature Review,” International Journal of Burns and Trauma 14, no. 5 (2024): 101–106. 39583335 10.62347/FZKH6176 PMC11579402

[15] L. Liu , Q. Gui , F. Zhao , X.‐z. Shen , and Y.‐l. Pei , “Isolated Partial Femoral Avulsion Fracture of the Posterior Cruciate Ligament in Adults,” Orthopaedic Surgery 13, no. 4 (2021): 1290–1298. 33960134 10.1111/os.12951 PMC8274204

[16] E. A. White , D. B. Patel , G. R. Matcuk , et al., “Cruciate Ligament Avulsion Fractures: Anatomy, Biomechanics, Injury Patterns, and Approach to Management,” Emergency Radiology 20, no. 5 (2013): 429–440. 23525909 10.1007/s10140-013-1121-0

[17] M. H. Meyers and F. M. McKeever , “Fracture of the Intercondylar Eminence of the Tibia,” Journal of Bone and Joint Surgery 41, no. 2 (1959): 209–222. 13630956

[18] P. O. Hooper, III , C. Silko , T. L. Malcolm , and L. D. Farrow , “Management of Posterior Cruciate Ligament Tibial Avulsion Injuries: A Systematic Review,” American Journal of Sports Medicine 46, no. 3 (2018): 734–742. 28437619 10.1177/0363546517701911

[19] G. K. Ren , Y. H. Tian , M. Y. Cui , et al., “Three‐Dimensional Classification and Clinical Treatment of Posterior Cruciate Ligament Tibial Avulsion Fracture Based on CT,” Zhong Guo Gu Shang=China Journal of Orthopaedics and Traumatology 38, no. 4 (2025): 389–395. 10.12200/j.issn.1003-0034.20240639 40296601

[20] M. S. Schulz , K. Russe , A. Weiler , H. J. Eichhorn , and M. J. Strobel , “Epidemiology of Posterior Cruciate Ligament Injuries,” Archives of Orthopaedic and Trauma Surgery 123 (2003): 186–191. 12734718 10.1007/s00402-002-0471-y

[21] M. Schlumberger , P. Schuster , M. Eichinger , et al., “Posterior Cruciate Ligament Lesions Are Mainly Present as Combined Lesions Even in Sports Injuries,” Knee Surgery, Sports Traumatology, Arthroscopy 28, no. 7 (2020): 2091–2098. 10.1007/s00167-020-05919-4 32157362

[22] N. A. Trasolini , G. F. Hatch , D. Wright , et al., “Posterior Cruciate Ligament Reconstruction With Internal Brace Augmentation Reduces Posterior Tibial Translation Under Cyclic Loading,” Orthopedics 44, no. 4 (2021): 235–240. 34292810 10.3928/01477447-20210621-03

[23] A. Otto , A. Helal , F. B. Imhoff , et al., “Promising Clinical and Magnetic Resonance Imaging Results After Internal Bracing of Acute Posterior Cruciate Ligament Lesions in Multiple Injured Knees,” Knee Surgery, Sports Traumatology, Arthroscopy 28 (2020): 2543–2550. 10.1007/s00167-020-05852-6 32047998

[24] J. A. Grotting , T. J. Nelson , M. B. Banffy , et al., “Biomechanical Evaluation of PCL Reconstruction With Suture Augmentation,” Knee 27, no. 2 (2020): 375–383. 32014412 10.1016/j.knee.2020.01.004

[25] B. Chernchujit and A. Situmeang , “Arthroscopic Fixation of Posterior Cruciate Ligament Avulsion Fracture With Combined Suture Tape and Internal Bracing by Suture‐Bridge Technique,” Orthopaedic Journal of Sports Medicine 11, no. 2_suppl (2023): 2325967121S00877.

[26] O. Hapa , F. A. Barber , G. Süner , et al., “Biomechanical Comparison of Tibial Eminence Fracture Fixation With High‐Strength Suture, EndoButton, and Suture Anchor,” Arthroscopy: The Journal of Arthroscopic & Related Surgery 28, no. 5 (2012): 681–687. 22284410 10.1016/j.arthro.2011.10.026

[27] J. Zhao , Y. He , and J. Wang , “Arthroscopic Treatment of Acute Tibial Avulsion Fracture of the Posterior Cruciate Ligament With Suture Fixation Technique Through Y‐Shaped Bone Tunnels,” Arthroscopy: The Journal of Arthroscopic & Related Surgery 22, no. 2 (2006): 172–181. 16458803 10.1016/j.arthro.2005.10.020

[28] A. Goyal , M. Tanwar , D. Joshi , and D. Chaudhary , “Practice Guidelines for the Management of Multiligamentous Injuries of the Knee,” Indian Journal of Orthopaedics 51 (2017): 537–544. 28966377 10.4103/ortho.IJOrtho_228_17 PMC5609375

[29] P. Zhou , J. Liu , Y. Xu , D. Wei , X. Deng , and Z. Li , “Early Effectiveness of Minimally Invasive Open Reduction and Internal Fixation Versus Arthroscopic Double‐Tunnel Suture Fixation for Tibial Avulsion Fracture of Posterior Cruciate Ligament,” Chinese Journal of Reparative and Reconstructive Surgery 34, no. 6 (2020): 707–712. 32538560 10.7507/1002-1892.201911049 PMC8171530

[30] Y. Qi , Y. T. Wang , F. M. Li , et al., “Treatment of Avulsion Fracture of Tibial Insertion Point of Posterior Cruciate Ligament by Modified Posterior Approach of Knee Joint,” Journal of Practical Orthopaedics 23, no. 3 (2017): 273–275.

[31] L. Willinger , L. Lacheta , C. von Deimmling , J. Lang , A. Imhoff , and P. Forkel , “Suture‐Bridge Technique for Tibial Avulsion Fractures of the Posterior Cruciate Ligament—A Biomechanical Comparison,” Orthopaedic Journal of Sports Medicine 7, no. 6_suppl4 (2019): 2325967119S00226. 10.1007/s00402-019-03278-5 31559489

[32] S. Ergün , U. Akgün , F. A. Barber , and M. Karahan , “The Clinical and Biomechanical Performance of All‐Suture Anchors: A Systematic Review,” Arthroscopy, Sports Medicine, and Rehabilitation 2, no. 3 (2020): e263–e275. 32548592 10.1016/j.asmr.2020.02.007 PMC7283965

[33] Y. Bozkurt and E. Karayel , “3D Printing Technology; Methods, Biomedical Applications, Future Opportunities and Trends,” Journal of Materials Research and Technology 14 (2021): 1430–1450.

[34] A. Boretti , “A Perspective on 3D Printing in the Medical Field,” Annals of 3D Printed Medicine 13 (2024): 100138.

[35] Z. Qu , J. Yue , N. Song , and S. Li , “Innovations in 3D Printed Individualized Bone Prosthesis Materials: Revolutionizing Orthopedic Surgery: A Review,” International Journal of Surgery 110 (2024): 6748–6762, 10.1097/JS9.0000000000001842. 38905508 PMC11486933

[36] R. Singla , A. Devgan , P. Gogna , and A. Batra , “Fixation of Delayed Union or Non‐Union Posterior Cruciate Ligament Avulsion Fractures,” Journal of Orthopaedic Surgery 22, no. 1 (2014): 70–74. 24781618 10.1177/230949901402200118

[37] H. Seitz , I. Schlenz , G. Pajenda , and V. Vécsei , “Tibial Avulsion Fracture of the Posterior Cruciate Ligament: K‐Wire or Screw Fixation? A Retrospective Study of 26 Patients,” Archives of Orthopaedic and Trauma Surgery 116 (1997): 275–278. 9177803 10.1007/BF00390052

